# Transgenic *Forsythia* plants expressing sesame cytochrome P450 produce beneficial lignans

**DOI:** 10.1038/s41598-022-14401-9

**Published:** 2022-06-16

**Authors:** Tomotsugu Koyama, Erika Matsumoto, Toshimi Okuda, Jun Murata, Manabu Horikawa, Naoki Hata, Atsushi Okazawa, Eiichiro Ono, Honoo Satake

**Affiliations:** 1 Bioorganic Research Institute, Suntory Foundation for Life Sciences, Seikacho, Kyoto 619-0284 Japan; 2 Department of Biological Resources Management, School of Environmental Science, The University of Shiga Prefecture, 2500 Hassaka-cho, Hikone, Shiga 522-8533 Japan; 3 Graduate School of Life and Environmental Sciences, Osaka Prefecture University, 1-1 Gakuen-cho, Naka-ku, Sakai, 599-8531 Japan; 4 Suntory Global Innovation Center (SIC) Ltd., Research Institute, Soraku-gun, Kyoto, 619-0284 Japan

**Keywords:** Biotechnology, Plant sciences

## Abstract

Lignans are widely distributed plant secondary metabolites that have received attention for their benefits to human health. Sesamin is a furofran lignan that is conventionally extracted from *Sesamum* seeds and shows anti-oxidant and anti-inflammatory activities in the human liver. Sesamin is biosynthesized by the *Sesamum*-specific enzyme CYP81Q1, and the natural sources of sesamin are annual plants that are at risk from climate change. In contrast, *Forsythia* species are widely distributed perennial woody plants that highly accumulate the precursor lignan pinoresinol. To sustainably supply sesamin, we developed a transformation method for *Forsythia* leaf explants and generated transgenic *Forsythia* plants that heterologously expressed the *CYP81Q1* gene. High-performance liquid chromatography (HPLC) and LC-mass spectrometry analyses detected sesamin and its intermediate piperitol in the leaves of two independent transgenic lines of *F. intermedia* and *F. koreana*. We also detected the accumulation of sesamin and piperitol in their vegetatively propagated descendants, demonstrating the stable and efficient production of these lignans. These results indicate that CYP81Q1-transgenic *Forsythia* plants are promising prototypes to produce diverse lignans and provide an important strategy for the cost-effective and scalable production of lignans.

## Introduction

The aging of world populations highlights the importance of plant secondary metabolites such as alkaloids, flavonoids, terpenoids, and lignans with benefits for human health^1,2^. Lignans are phenylpropanoid dimers with diverse functions, and dietary lignans have attracted attention as food nutrients^3,4^. (+)-Sesamin is a furofuran lignan that is commercially available as a health-promoting supplement^5^. In mammals, (+)-Sesamin metabolites attenuate oxidation and inflammation for the protection of the liver^6,7^. (+)-Sesamin also shows anti-cancer properties^8^. (+)-Sesamin is commercially available via extraction at concentrations (4–6 mg/g) from *Sesamum indicum* (sesame) seed oil^5,9,10^. Sesame plants, the strongest known synthesizers of (+)-sesamin, are annuals that are threatened by climate change^11,12^. Thus, new plant sources are required for the efficient and stable production of (+)-sesamin.

**Figure 1:**
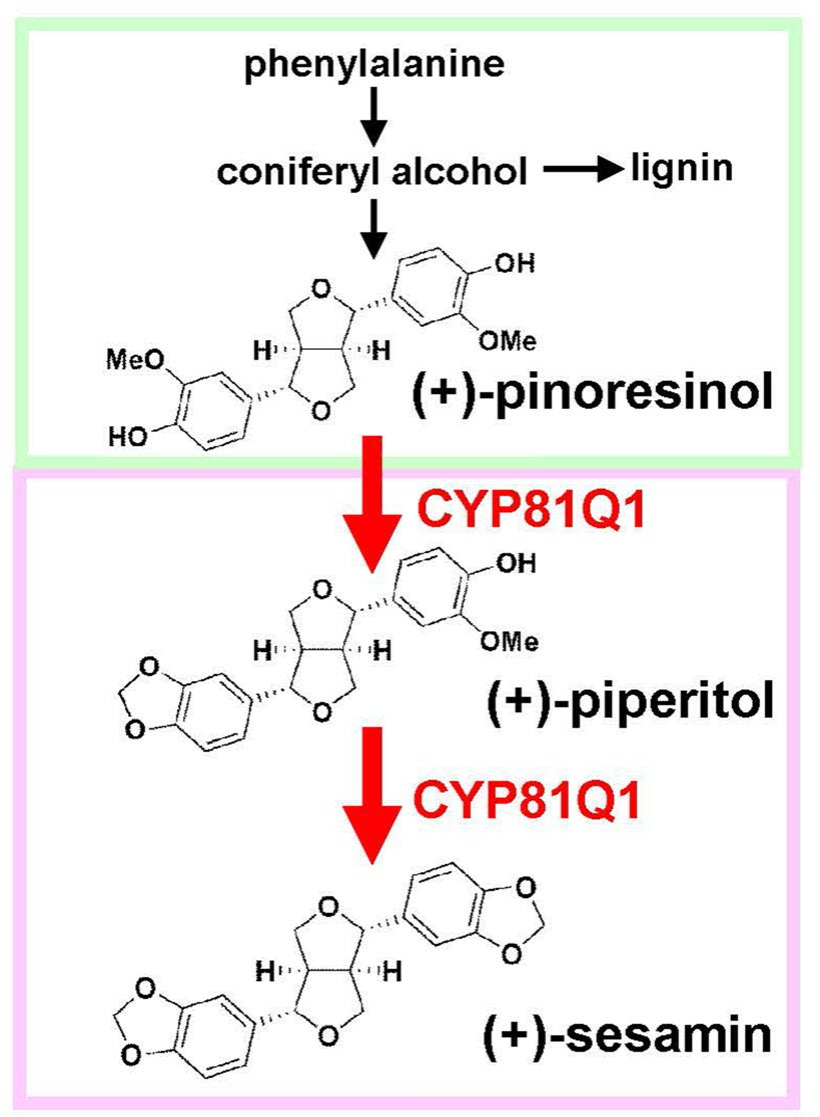
Biosynthesis of (+)-piperitol and (+)-sesamin in *Forsythia* plants by heterologous expression of the sesame *CYP81Q1* gene. Lignan biosynthesis is initiated from phenylalanine. Two coniferyl alcohols are coupled to the precursor lignan (+)-pinoresinol in land plants (green). In sesame plants, CYP81Q1 sequentially synthesizes (+)-piperitol and (+)-sesamin (red).

In land plants, the lignan metabolic pathway branches from that of lignin at the coupling of monolignols such as coniferyl alcohol, which is synthesized from phenylalanine (Fig. 1)^13,14^. Dirigent protein metabolizes coniferyl alcohol and specifically synthesizes the precursor lignan pinoresinol (Fig. 1)^15–18^. The lignan metabolic pathway further diverges into structurally and functionally diverse lignans by plant species-specific enzymes (Fig. 1)^5,14,19^. In sesame plants, cytochrome P450 81Q1 (CYP81Q1) catalyzes the sequential conversion of (+)-pinoresinol to (+)-piperitol and (+)-sesamin (Fig. 1)^9^.

*Forsythia* species such as *Forsythia intermedia (Fi)* and *F. koreana (Fk)* are widely distributed perennial woody plants. Extracts of *Forsythia* plants have been empirically used in traditional medicines^20^. *Forsythia* species produce various lignans and other polyphenols^21–23^, and their biosynthesis and regulation have been analyzed^18,20,21,24,25^. *Forsythia* leaves accumulate the precursor lignan (+)-pinoresinol at high levels but lack (+)-sesamin biosynthesis (Fig. 1)^21^. To sustainably supply beneficial lignans including (+)-sesamin, heterologous expression of the lignan-biosynthetic enzyme genes in *Forsythia* plants is promising.

Previously, we demonstrated the ectopic accumulation of (+)-sesamin in cultured *CYP81Q1*-transgenic *Fk* cells^26,27^ and showed their ability to produce (+)-sesamin. However, the mass production of (+)-sesamin using transgenic cells is not practical in light of the cost of large-scale cell culture. In contrast, given the large biomass generated by *Forsythia* leaves, *CYP81Q*-transgenic *Forsythia* plants could efficiently and stably produce (+)-sesamin. In this study, we generated *CYP81Q1*-transgenic *Forsythia* plants that stably produce the intermediate (+)-piperitol and the product (+)-sesamin.

## Results

**Figure 2:**
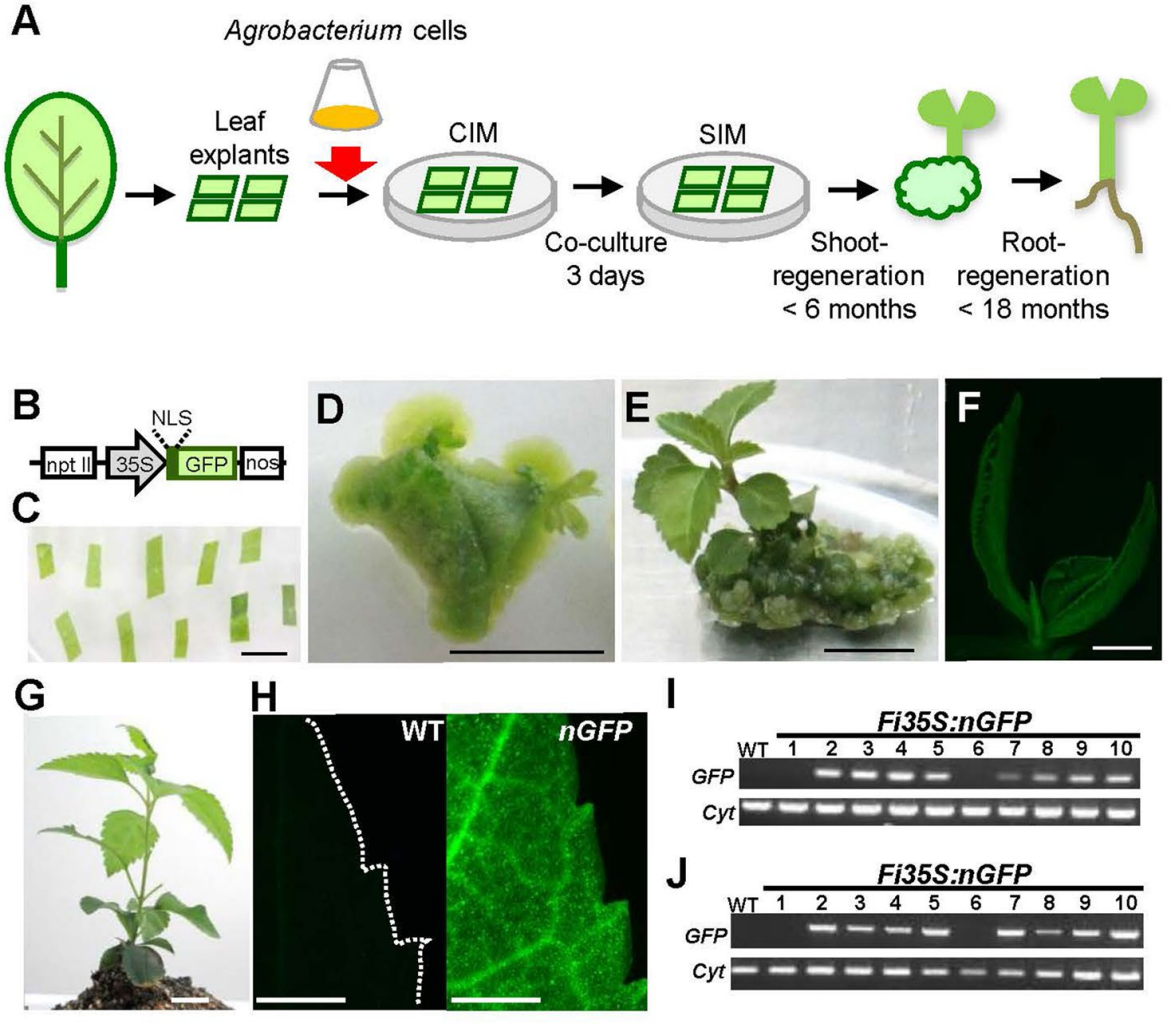
Generation of transgenic *Pro35S:nGFP* plants. (**A**) A scheme of *Agrobacterium*-mediated transformation of *Forsythia* leaf explants followed by the regeneration of the whole plant body. *CIM* callus-inducing medium, *SIM* shoot-inducing medium. (**B**) The *Pro35S:nGFP* plasmid. *nptII* kanamycin resistance gene, *35S* cauliflower mosaic virus 35S promoter, *NLS* nuclear localization signal, *GFP* green florescence protein, *nos* nos terminator. (**C**) Leaf explants co-cultured with *Agrobacterium* cells. (**D**) Leaf explants dedifferentiated into calluses and regenerating adventitious shoots. (**E**) An adventitious shoot before separation from calluses. (**F**) GFP fluorescence in a *FiPro35S:nGFP* shoot. (**G**) A *FiPro35S:nGFP* plant transferred into soil. (**H**) GFP fluorescence in a *FiPro35S:nGFP* leaf (*nGFP*; right) in contrast with the lack of fluorescence in a non-transformed wild-type (WT) leaf (left). Dotted white line marks the WT leaf margin. (**I**, **J**) Genomic (**I**) and reverse transcription-polymerase chain reaction (RT-PCR) (**J**) analyses of *GFP* gene. *Cyt* (cytochrome) served as an internal control. The numbers above the gel images indicate the plant lines regenerated from independent calluses. Scale bars = 1 cm in (**C**) to (**H**).

Initially, we established a practical method for the transformation of *Forsythia* plants (Fig. 2A). *Fi* leaf explants (n = 451) were soaked in a suspension of *Agrobacterium tumefaciens* cells harboring the *Pro35S:nGFP*^28^ plasmid and regenerated shoots and roots during culture for two years (see “Materials and methods”, Fig. 2B–G, Table 1). Eight of ten independent kanamycin-resistant lines exhibited signals for *nGFP* presence and expression when examined by fluorescence microscopy (Fig. 2H) and genomic (Fig. 2I) and RT-PCR analyses (Fig. 2J); no *nGFP* presence or expression was seen in wild-type (WT) plants. Two kanamycin-resistant plants did not show GFP presence or expression, probably due to somaclonal variation that eliminated the transgene (Fig. 2I,J). Even after vegetative propagation for four years, newly generated *FiPro35S:nGFP* leaves maintained GFP fluorescence, demonstrating stable *Pro35S:nGFP* transformation of *Forsythia* plants.

**Table 1:** Summary on the generation of transgenic *Forsythia* plants.

Plant species	Gene transformed	Leaf explants	Transgenic plants
*Forsythia intermedia*	*Pro35S:nGFP*	451	8^a^
*Forsythia intermedia*	*Pro35S:CYP81Q1*	956	2^b^
*Forsythia koreana*	*Pro35S:CYP81Q1*	273	2^b^

^a^Kanamycin-resistant plants presenting GFP fluorescence.

^b^Kanamycin-resistant plants presenting expression of the *CYP81Q1* gene in RT-PCR analysis.

**Figure 3:**
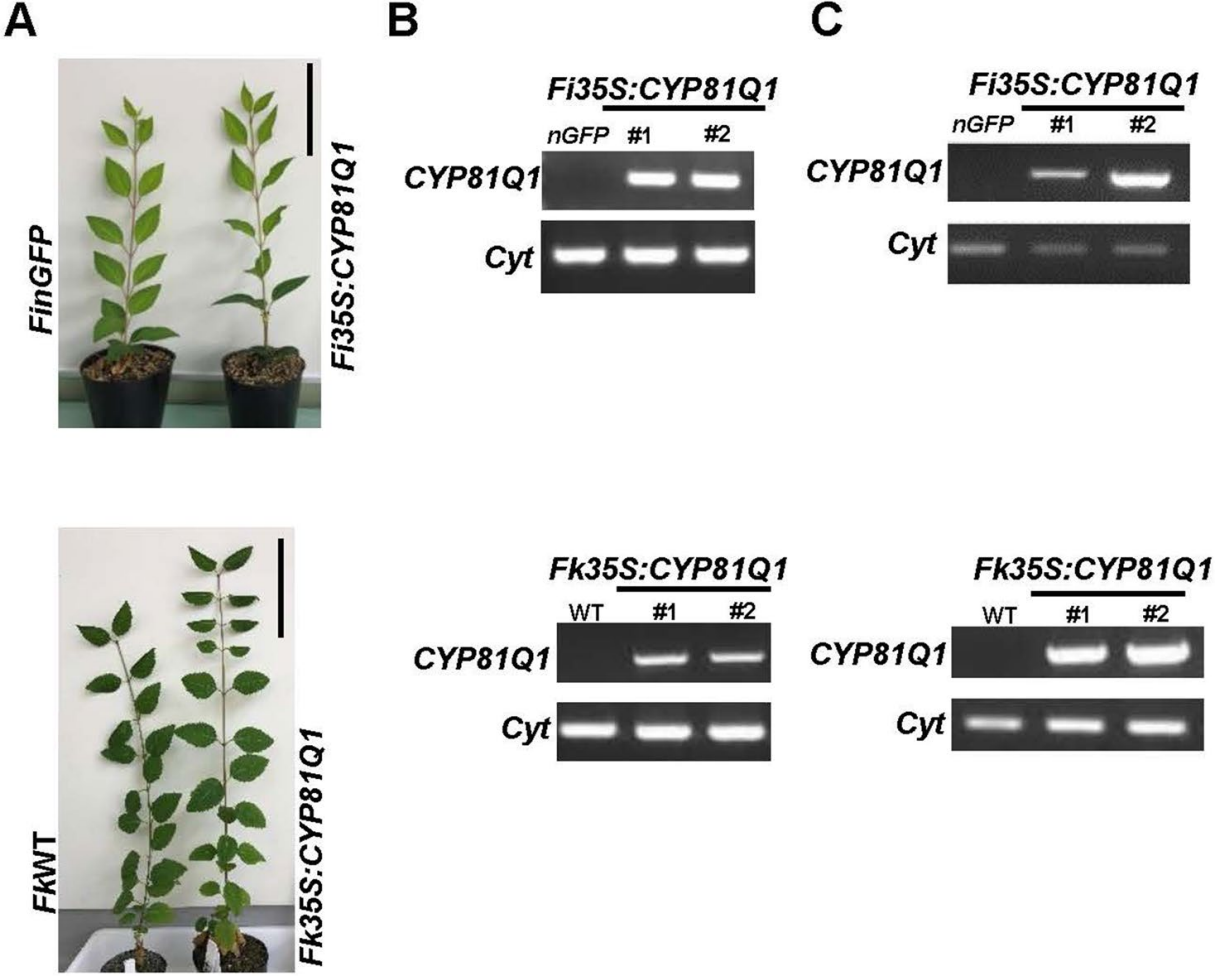
Generation of transgenic *Pro35S:CYP81Q1* plants. (**A**) Control and *Pro35S:CYP81Q1* plants. Scale bars = 10 cm. (**B**, **C**) Genomic (**B**) and reverse transcribed (RT)-PCR (**C**) analyses of *CYP81Q1* gene. *Cyt* (cytochrome) served as an internal control. The numbers above the gel images indicate the plant lines regenerated from independent calluses.

We also introduced the 35*S* promoter-regulated sesame *CYP81Q1* gene (*Pro35S:CYP81Q1*)^29^ into *Fi* and *Fk*. After co-culture of 956 *Fi* and 273 *Fk* leaf explants with *Agrobacterium* cells harboring the *Pro35S:CYP81Q1* plasmid, the resulting transgenic *Forsythia* plants were propagated vegetatively in soil through repeated rounds of cutting and growth in our plant culture room—conditions under which *Forsythia* plants continuously developed their leaves without flowering (Table 1). Eventually, two independent transgenic lines developed normally on soil (Fig. 3A) and showed *CYP81Q1* gene presence (Fig. 3B) and expression (Fig. 3C).

**Figure 4:**
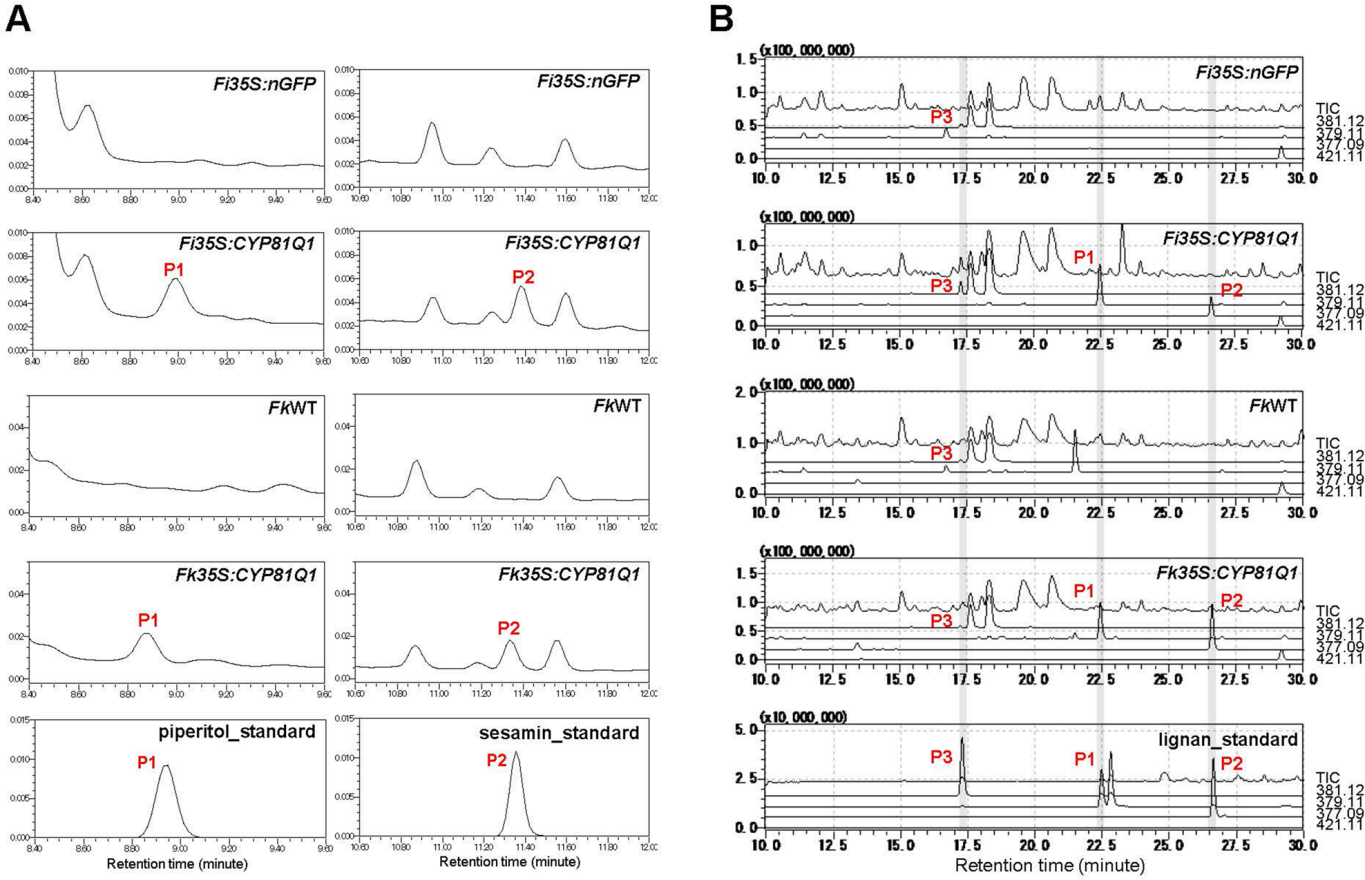
HPLC (**A**) and LC–MS (**B**) analysis of control and *Pro35S:CYP81Q1* plants. Chromatograms showing specific accumulation of (+)-piperitol (P1) and (+)-sesamin (P2) in the *Pro35S:CYP81Q1* leaves. In (**B**), pinoresinol (P3); m/z = 381.12, piperitol; m/z = 379.11, sesamin; m/z = 377.09, 3′-ethoxysesamin; m/z = 421.11, *TIC* total ion current.

To detect the accumulation of the products of CYP81Q1, we subjected the leaves of *FiPro35S:CYP81Q1, FkPro35S:CYP81Q1,* control *FiPro35S:nGFP,* and *FkWT* plants to high-performance liquid chromatography (HPLC) and LC-mass spectrometry (MS) analyses (Fig. 4). HPLC and LC–MS analyses indicated that the heterologous expression of the *CYP81Q1* gene in *Forsythia* leaves resulted in the production of (+)-piperitol and (+)-sesamin (Fig. 4), and that the leaves of the primary transformants *FiPro35S:CYP81Q1* and *FkPro35S:CYP81Q1* accumulated (+)-piperitol (14.23 and 39.45 µg/g DW, respectively) and (+)-sesamin (5.57 and 27.21 µg/g DW), while control *FiPro35S:nGFP* and *Fk*WT plants did not (Table 2).

**Table 2:** The accumulation of (+)-piperitol and (+)-sesamin in *Pro35S:CYP81Q1* leaves.

Genotype	Piperitol (µg/gDW)	Sesamin (µg/gDW)	Piperitol + sesamin (µg/gDW)
*FinGFP* (n = 1) primary transformant	n.d.	n.d.	n.d.
*FiCYP81Q1* (n = 1) primary transformant	14.23	5.57	19.80
*FinGFP* (n = 6) 2nd propagation	n.d.	n.d.	n.d.
*FiCYP81Q1* (n = 6) 2nd propagation	39.57** (SD ± 25.26)	11.09* (SD ± 11.89)	50.66
*FinGFP* (n = 5) 3rd propagation	n.d.	n.d.	n.d.
*FiCYP81Q1* (n = 5) 3rd propagation	17.81* (SD ± 15.76)	1.69* (SD ± 1.60)	19.50
*Fk*WT (n = 1) primary plant	n.d.	n.d.	n.d.
*FkCYP81Q1* (n = 1) primary transformant	39.45	27.21	66.65
*Fk*WT (n = 6) 2nd propagation	n.d.	n.d.	n.d.
*FkCYP81Q1* (n = 6) 2nd propagation	34.39** (SD ± 14.24)	19.45** (SD ± 17.17)	53.84
*Fk*WT (n = 6) 3rd propagation	n.d.	n.d.	n.d.
*FkCYP81Q1* (n = 6) 3rd propagation	51.66** (SD ± 16.48)	21.50** (SD ± 8.34)	73.16

*n.d.* contents below detectable levels.

*SD* standard deviation of six biological replicates.

*P < 0.05, **P < 0.01; mean values were significantly different from those of the control by two-tailed Student’s *t* test.

We further examined whether the ability to biosynthesize piperitol and sesamin was inherited by descendant transgenic plants. After two and three rounds of vegetative propagation, descendant *Pro35S:CYP81Q1* plants produced similar amounts of (+)-piperitol and (+)-sesamin (Table 2). The total amounts of (+)-piperitol and (+)-sesamin (Table 2) exceeded those in *CYP81Q1-Fk* cells (10 µg/g DW)^5^. In contrast, the LC–MS analysis detected pinoresinol (P3 in Fig. 4B) and the content of pinoresinol was not changed in the control and the transgenic *Pro35S:CYP81Q1* plants [(*FiPro35S:nGFP* and *FiPro35S:CYP81Q1* (9.34 ± 3.64 and 11.68 ± 5.44 mg/g DW, respectively) and *FkWT* and *FkPro35S:CYP81Q1* (24.24 ± 10.06 and 25.35 ± 7.63 mg/g DW, respectively)]; Supplemental Fig. S1), indicating that the biosynthesis of pinoresinol is intact in the presence of CYP81Q1. Altogether, we demonstrated that the *Pro35S:CYP81Q1* plants efficiently produced (+)-piperitol and (+)-sesamin.

## Discussion

In this study, we provide evidence that transgenic *Forsythia* plants produce ectopic lignans, (+)-piperitol and (+)-sesamin. These results suggest that sesamolin and sesaminol, which are antioxidant sesame lignans metabolized from (+)-sesamin by CYP92B14^30^, is expected to be produced via additional introduction of the *CYP92B14* gene into *Pro35S:CYP81Q1* plants. Another lignan, podophyllotoxin, may also be produced in transgenic *Forsythia* plants. Podophyllotoxin is at present extracted from the rhizomes of *Podophyllum* species and clinically utilized in cancer therapy^31^. In *Podophyllum* podophyllotoxin biosynthesis, matairesinol, which *Forsythia* species also accumulate in the pathway downstream of pinoresinol, is metabolized to pluviatolide by CYP719A23^32^. Additional enzymes (CYP71CU1, 2-oxoglutarate/Fe(II)-dependent dioxygenase, and *O*-methyltransferases) convert pluviatolide to the proposed precursor deoxypodophyllotoxin^33^. Thus, methods for introducing multiple genes into *Forsythia* plants will pave the way for the generation of podophyllotoxin and its related compounds by transgenic plants. In a previous study, we generated triple-transgenic *Forsythia* cultured cells^26^, suggesting the possibility of multigene transformation of *Forsythia* plants.

The production of various specialized plant metabolites has been attempted in transgenic and synthetic biology-based microorganisms^1,34–40^. However, most microorganisms appear to lack (+)-sesamin and its precursor (+)-pinoresinol^9,41^. Moreover, tremendous bioinformatic and screening processes are often required for the generation of genetically-engineered microorganisms that produce plant lignans. Additionally, mass production using such engineered microorganisms could be limited by genetic instability, infectious contamination, unexpected product toxicity, and low fermentation performance^38,40^. In contrast, *Forsythia* species are perennial shrubs, and thus *Pro35S:CYP81Q1* plants are easily cultivated via explant, a marked advantage for stable growth and mass propagation in a plant factory. Moreover, a plant factory provides plasticity in place and time of the production of sesamin in plants^42^, unlike agricultural production that is limited by climates, seasons, and farmlands. Thus, we will be able to produce sesamin using our transgenic *Forsythia* plants in a plant factory.

Also of significance is the reproducible generation of transgenic *Forsythia* plants expressing the *CYP81Q1* gene. The lignan biosynthetic pathway has previously been modified in cultured plants, cells, and hairy roots of *Carthamus*, *Linum*, *Hyptis*, *Juniperus*, *Podophyllum*, *Sesamum*, and *Forsythia* species^43–59^, but the transgenic plants have not been reported. For the scalable production of lignans, our transgenic *Forsythia* plant method provides an important strategy for future metabolic engineering of such lignan-producing plants.

The transgenic *Forsythia* plants have limitation in the content of sesamin as compared with sesame seeds. To increase the content of sesamin in the transgenic *Forsythia* plants, we will be able to apply multigene transformation strategy. Previously, triple-transgenic *Forsythia* cells with the RNAi construct for endogenous pinoresinol-lariciresinol reductase, and the overexpression-construct of pinoresinol glucosylating enzyme, which increase the level of the precursor pinoresinol, as well as *CYP81Q1* gene, produced higher level of sesamin than the single-transgenic *CYP81Q1* cells^26^. The same strategy of multigene transformation of *Forsythia* plants is expected to increase the content of sesamin. Moreover, overexpression of enzymes upstream of pinoresinol stimulated accumulation of podophyllotoxin-related lignans^60^. Thus, overexpression of the upstream enzymes may increase the content of sesamin in transgenic *Forsythia* plants. Because the content of pinoresinol was not changed in the control and the *Pro35S:CYP81Q1* plants (Supplemental Fig. S1), the moderate activity of CYP81Q1 seems to limit the rate of production of sesamin from pinoresinol. As many cytochrome P450 enzymes function in complex with their native oxidoreductases^30^, co-expression of *CYP81Q1* and *S. indicum* oxidoreductase genes in transgenic *Forsythia* plants is a future option to strengthen the activity of CYP81Q1 for higher production of sesamin. In addition, the red light condition increased the content of sesamin in the transgenic *Forsythia* cells^26^, suggesting that irradiation of red light to transgenic *Forsythia* plant may increase the content of sesamin.

In conclusion, we have generated *Forsythia* plants as promising prototypes for the efficient and sustainable heterologous production of beneficial lignans.

## Materials and methods

### Plant growth conditions

*F. intermedia* and *F. koreana* plants were obtained from Niigata Prefectural Botanical Garden and Dr. Toshiaki Umezawa (Kyoto University), respectively. The use of these plants for this study has been approved by the providers. All the experimental work on plant material described in this study complies with the relevant institutional, national, and international guidelines and legislation. Both of *Forsythia* plants were maintained in pots filled with soil inside a plant culture room under a cycle of 16-h-light (photosynthetic photon flux density of 50 to 75 µmol m^−2^ s^−1^)/8-h-dark at 22 °C unless otherwise indicated. After surface-sterilization of 10 cm length *Forsythia* shoots using 70% ethanol for 30 s and 1% sodium hypochlorite solution for 15 min, the *Forsythia* plants were maintained in vitro in a culturing box on modified MS medium (MS salts, MS vitamin, 30 g/L sucrose, 22.5 mg/L CuSO_4_5H_2_O, 5 g/L Agargel [Sigma-Aldrich, MO]) and transferred to fresh medium every two to three months.

### Transformation and preparation of *Agrobacterium* cells

For construction of *Pro35S:nGFP*^28^, the *NLS* was inserted upstream of *GFP* and the resulting fusion *NLS-GFP* genes was inserted into pBI101. For construction of *Pro35S:CYP81Q1*^29^, the coding sequence of *CYP81Q1* (accession number AB194714) was inserted downstream of the *Pro35S* promoter in pBINplus. *Agrobacterium tumefaciens* GV3101 cells were individually transformed with *Pro35S:nGFP*^28^ and *Pro35S:CYP81Q1*^29^ plasmids using a Gene Pulser II electroporator (BioRad, CA). The resultant *Agrobacterium* cells were cultured in LB liquid medium at 27 °C until the optical density at 600 nm (OD600) reached 1.5 to 2.0. The *Agrobacterium* cells were collected by centrifugation of the medium (HP25; Beckman, CA) at 6000×*g* for 15 min at room temperature, resuspended in the transformation solution (Gamborg’s B5 salts, MS vitamins, 30 g/L sucrose, 2.0 mg/L indole-3-acetic acid [IAA; Nacalai tesque, Japan], 0.5 mg/L 6-benzyladenopurine [BA; Nacalai tesque, Japan], 20 mg/L acetosyringone [Tokyo Chemical Industry, Japan], 0.02% 500 W Additive [equivalent to Silwet L-77; DOW CORNING, MI]), and further diluted with additional transformation solution to a concentration of OD600 = 0.7.

### Transformation and culture of *Forsythia* leaf explants

To prepare leaf explants, the third to sixth leaves from the top of one-month-cultured *Forsythia* plants were detached at their petioles and cut into 10 × 5 mm squares using a surgical knife. For the co-culture, the leaf explants were submerged in the *Agrobacterium* cell-suspended transformation solution for 2 min at room temperature, the transformation solution was wiped off using sterilized paper towels, and the explants were transferred onto sterilized filter papers (No.1 70 mm; ADVANTEC, Japan) over callus-inducing medium (CIM; Gamborg’s B5 salts, B5 vitamin, 30 g/L sucrose, 2.0 mg/L IAA, 0.5 mg/L BA, 20 mg/L acetosyringone, 3 g/L gellan gum [Kanto Chemical, Japan]), and maintained at 22 °C in the dark for three days. To suppress the overgrowth of the *Agrobacterium* cells, the leaf explants were transferred onto shoot-inducing medium (SIM; MS salts, MS vitamin, 30 g/L sucrose, 0.5 mg/L IAA, 2.0 mg/L BA, 3 g/L gellan gum) supplemented with 10 mg/L meropenem (Tokyo Chemical Industry, Japan) for four days in the dark. To isolate kanamycin-resistant plants, leaf explants were cultured on SIM supplemented with kanamycin (75 mg/L for *F. intermedia* and 50 mg/L for *F. koreana*; Nacalai tesque, Japan) and 10 mg/L meropenem under 16-h-light/8-h-dark conditions and transferred onto fresh medium every two weeks up to six months. Adventitious shoots regenerated at the periphery of the dedifferentiated explants were transferred onto shoot-elongating medium (MS salts, MS vitamin, 30 g/L sucrose, 22.5 mg/L CuSO_4_5H_2_O, 2.0 mg/L BA, 5 g/L Agargel) supplemented with kanamycin and meropenem in sterilized glass tubes until the shoots elongated to 5 cm height. Shoots were transferred and maintained on hormone-free medium (MS salts, MS vitamin, 30 g/L sucrose, 22.5 mg/L CuSO_4_·5H_2_O, 5 g/L Agargel) until their rooting.

### Microscopy

GFP fluorescence was observed using a M205 fluorescence stereomicroscope (Leica Microsystems, Germany) using the GFP3 filter and recorded by LAS AF software (Leica Microsystems, Germany).

### Extraction of nucleotides and detection of genes

The genomic DNAs of *Forsythia* leaves were prepared using a Nucleon PhytoPure Genomic DNA Extraction kit (GE Healthcare, Sweden) according to the manufacturer’s instruction. The total RNAs of *Forsythia* leaves were prepared using an RNAeasy Plant Mini Kit (Qiagen, Germany) according to the manufacturer’s instruction, cleared by precipitation in 4 M lithium chloride solution (final concentration), treated with DNase I (Qiagen, Germany), and further subjected to reverse transcription using SuperScript III (Thermo Fisher Scientific, MA) with an Oligo(dT) primer (Thermo Fisher Scientific, MA). For the detection of genes of interest, the genomic and complementary DNAs were individually subjected to PCR analysis using appropriate sets of primers (Supplementary Table S1), electrophoresed, and stained by ethidium bromide solution (Supplementary Fig. S2).

### Measurement of lignans

Cultured transgenic and control *Forsythia* plants were transferred into soil in pots, acclimatized for three weeks until rooting, and grown for two months until the plants reached 15 to 20 cm height. The third to fifth leaves from the top of each plant were pooled, frozen in liquid nitrogen, lyophilized to permit measurement of dry weight (DW) using an FDU-2110 device (EYELA, Japan), extracted with 50% methanol (v/v) containing 2.25 µM (final concentration) 2′-ethoxysesamin (Supplementary Fig. S3 and Table S2) as the internal standard, and processed as described previously^29^. The leaf extracts were subjected into reverse-phase HPLC (Alliance 2960, Waters Corporation, MA) using a Develosil C30-UG-5 column (4.6 × 150 mm; Nomura Chemical, Japan) under conditions described previously^26^. Lignans were monitored by UV absorption at 283 nm, and their concentrations were calculated by Empower2 software (https://www.waters.com/waters/library.htm?locale=en_US&lid=1529008,Waters Corporation, MA) according to the areas of the peaks in the chromatograms while referencing standard curves of authentic piperitol and sesamin with technical duplicates or triplicates. For LC–MS analysis, the leaf extracts were subjected to LC–MS-IT-TOF (Shimazu, Japan) and analyzed as described previously^30^. Lignans were detected using a photodiode array detector and and analyzed by LabSolutions LCMS version 3.8.1 (https://www.an.shimadzu.co.jp/lcms/support/download/index.htm, Shimazu, Japan).

### Statistical analysis

The lignan contents were measured using five or six biological replicates and statistically analyzed by two-tailed Student’s *t* tests (Table 2 and Supplemental Fig. S1).

## Supplementary Information

Supplementary Information.

## References

[1] Wurtzel ET, Kutchan TM (2016). Plant metabolism, the diverse chemistry set of the future. Science.

[2] Jacobowitz JR, Weng J-K (2020). Exploring uncharted territories of plant specialized metabolism in the postgenomic era. Ann. Rev. Plant Biol..

[3] Durazzo A (2018). Dietary lignans: Definition, description and research trends in databases development. Molecules.

[4] Rodríguez-García C, Sánchez-Quesada C, Toledo E, Delgado-Rodríguez M, Gaforio JJ (2019). Naturally lignan-rich foods: A dietary tool for health promotion?. Molecules.

[5] Satake H (2015). Essences in metabolic engineering of lignan biosynthesis. Metabolites.

[6] Kabe Y (2020). Annexin A1 accounts for an anti-inflammatory binding target of sesamin metabolites. NPJ Sci. Food.

[7] Nakai M (2003). Novel antioxidative metabolites in rat liver with ingested sesamin. J. Agric. Food Chem..

[8] Majdalawieh AF, Massri M, Nasrallah GK (2017). A comprehensive review on the anti-cancer properties and mechanisms of action of sesamin, a lignan in sesame seeds (*Sesamum indicum*). Eur. J. Pharm..

[9] Ono E (2006). Formation of two methylenedioxy bridges by a *Sesamum* CYP81Q protein yielding a furofuranlignan, (+)-sesamin. Proc. Natl. Acad. Sci. USA.

[10] Andargie M, Vinas M, Rathgeb A, Möller E, Karlovsky P (2021). Lignans of sesame (*Sesamum indicum*): A comprehensive review. Molecules.

[11] Dossa K (2017). The emerging oilseed crop sesamum indicum enters the “Omics” era. Front. Plant Sci..

[12] Dossa K (2019). The genetic basis of drought tolerance in the high oil crop *Sesamum indicum*. Plant Biotechnol. J..

[13] Davin LB, Lewis NG (2000). Dirigent proteins and dirigent sites explain the mystery of specificity of radical precursor coupling in lignan and lignin biosynthesis. Plant Physiol..

[14] Suzuki S, Umezawa T (2007). Biosynthesis of lignans and norlignans. J. Wood Sci..

[15] Kim KW (2015). Trimeric structure of (+)-pinoresinol-forming dirigent protein at 195 A° resolution with three isolated active sites. J. Biol. Chem..

[16] Yonekura-Sakakibara K (2021). Seed-coat protective neolignans are produced by the dirigent protein AtDP1 and the laccase AtLAC5 in Arabidopsis. Plant Cell.

[17] Gasper R (2016). Dirigent protein mode of action revealed by the crystal structure of AtDIR6. Plant Physiol..

[18] Davin LB (1997). Stereoselective bimolecular phenoxy radical coupling by an auxiliary (dirigent) protein without an active center. Science.

[19] Teponno RB, Kusari S, Spiteller M (2016). Recent advances in research on lignans and neolignans. Nat. Prod. Rep..

[20] Rosati C, Cadic A, Duron M, Simoneau P, Pua EC, Davey MR (2007). Forsythia. Biotechnology in Agriculture and Forestry. Transgenic Crops VI.

[21] Umezawa T (2003). Diversity in lignan biosynthesis. Phytochem. Rev..

[22] Guo H, Liu AH, Ye M, Yang M, Guo DA (2007). Characterization of phenolic compounds in the fruits of *Forsythia suspensa* by high-performance liquid chromatography coupled with electrospray ionization tandem mass spectrometry. Rap. Commun. Mass Spectrom..

[23] Dong Z (2017). *Forsythiae Fructus*: A review on its phytochemistry, quality control, pharmacology and pharmacokinetics. Molecules.

[24] Shiraishi A (2016). De novo transcriptomes of *Forsythia koreana* using a novel assembly method: Insight into tissue- and species-specific expression of lignan biosynthesis-related gene. PLoS ONE.

[25] Sun L (2018). Comparative transcriptome analyses of three medicinal *Forsythia* species and prediction of candidate genes involved in secondary metabolisms. J. Nat. Med..

[26] Murata J, Matsumoto E, Morimoto K, Koyama T, Satake H (2015). Generation of triple-transgenic *Forsythia* cell cultures as a platform for the efficient, stable, and sustainable production of lignans. PLoS ONE.

[27] Kim HJ (2009). Metabolic engineering of lignan biosynthesis in forsythia cell culture. Plant Cell Physiol..

[28] Koyama T (2013). A regulatory cascade involving class II ethylene response factor transcriptional repressors operates in the progression of leaf senescence. Plant Physiol..

[29] Tera M (2019). Identification of a binding protein for sesamin and characterization of its roles in plant growth. Sci. Rep..

[30] Murata J (2017). Oxidative rearrangement of (+)-sesamin by CYP92B14 co-generates twin dietary lignans in sesame. Nat. Commun..

[31] Gordaliza M (2007). Natural products as leads to anticancer drugs. Clin. Transl. Oncol..

[32] Marques JV (2013). Next generation sequencing in predicting gene function in podophyllotoxin biosynthesis. J. Biol. Chem..

[33] Lau W, Sattely ES (2015). Six enzymes from mayapple that complete the biosynthetic pathway to the etoposide aglycone. Science.

[34] Zhu Q (2020). Plant synthetic metabolic engineering for enhancing crop nutritional quality. Plant Commun..

[35] Sato F, Kumagai H (2013). Microbial production of isoquinoline alkaloids as plant secondary metabolites based on metabolic engineering research. Proc. Jpn. Acad. B..

[36] Kotopka BJ, Li Y, Smolke CD (2018). Synthetic biology strategies toward heterologous phytochemical production. Nat. Prod. Rep..

[37] Pyne ME, Narcross L, Martin VJJ (2019). Engineering plant secondary metabolism in microbial systems. Plant Physiol..

[38] Choi KR (2019). Systems metabolic engineering strategies: integrating systems and synthetic biology with metabolic engineering. Trends Biotechnol..

[39] Birchfield AS, McIntosh CA (2020). Metabolic engineering and synthetic biology of plant natural products: A minireview. Curr. Plant Biol..

[40] Arya SS, Rookes JE, Cahill DM, Lenka SK (2020). Next-generation metabolic engineering approaches towards development of plant cell suspension cultures as specialized metabolite producing biofactories. Biotech. Adv..

[41] Hano CF, Dinkova-Kostova AT, Davin LB, Cort JR, Lewis NG (2021). Lignans: Insights into their biosynthesis, metabolic engineering, analytical methods and health benefits. Front. Plant Sci..

[42] De Martinis D (2016). Plant molecular farming: Fast, scalable, cheap, sustainable. Front. Plant Sci..

[43] Fuss E (2003). Lignans in plant cell and organ cultures: An overview. Phytochem. Rev..

[44] Bayindir Ü, Alfermann AW, Fuss E (2008). Hinokinin biosynthesis in *Linum corymbulosum* Reichenb. Plant J..

[45] Ragamustari SK (2014). Substrate-enantiomer selectivity of matairesinol O-methyltransferases. Plant Biotechnol..

[46] Lalaleo L (2018). Effect of in vitro morphogenesis on the production of podophyllotoxin derivatives in callus cultures of *Linum album*. J. Plant Physiol..

[47] Renouard S (2018). Investigation of *Linum flavum* (L.) hairy root cultures for the production of anticancer aryltetralin lignans. Int. J. Mol. Sci..

[48] Bazaldúa C (2019). Improving the production of podophyllotoxin in hairy roots of *Hyptis suaveolens* induced from regenerated plantlets. PLoS ONE.

[49] Mikac S, Ramawat KG (2021). Bioproduction of anticancer podophyllotoxin and related aryltretralin-lignans in hairy root cultures of *Linum flavum* L. Reference Series in Phytochemistry. Plant Cell and Tissue Differentiation and Secondary Metabolites.

[50] Markulin L (2019). Pinoresinol–lariciresinol reductases, key to the lignan synthesis in plants. Planta.

[51] Muranaka T, Miyata K, Ito K, Tachibana S (1998). Production of podophyllotoxin in *Juniperus chinensis* callus cultures treated with oligosaccharides and a biogenetic precursor. Phytochemistry.

[52] Esmaeilzadeh Bahabadi S (2012). Time-course changes in fungal elicitor-induced lignan synthesis and expression of the relevant genes in cell cultures of *Linum album*. J. Plant Physiol..

[53] Bahabadi SE (2011). Increased lignan biosynthesis in the suspension cultures of *Linum album* by fungal extracts. Plant Biotechnol. Rep..

[54] Bahabadi ES (2014). The effect of chitosan and chitin oligomers on gene expression and lignans production in *Linum album* cell cultures. J. Med. Plants.

[55] Hazra S, Bhattacharyya D, Chattopadhyay S (2017). Methyl jasmonate regulates podophyllotoxin accumulation in *Podophyllum hexandrum* by altering the ROS-responsive podophyllotoxin pathway gene expression additionally through the down regulation of few interfering miRNAs. Front. Plant Sci..

[56] Corbin C (2017). Functional characterization of the pinoresinol–lariciresinol reductase-2 gene reveals its roles in yatein biosynthesis and flax defense response. Planta.

[57] Yousefzadi M (2012). The effect of light on gene expression and podophyllotoxin biosynthesis in *Linum album* cell culture. Plant Physiol. Biochem..

[58] Hata N (2013). Differences in plant growth and leaf sesamin content of the lignan-rich sesame variety “Gomazou” under continuous light of different wavelengths. Plant Biotechnol..

[59] Morimoto K (2011). Effects of light on production of endogenous and exogenous lignans by *Forsythia koreana* wildtype and transgenic cells. Plant Biotechnol..

[60] Shultz BJ, Kim SY, Lau W, Sattely ES (2019). Total biosynthesis for milligram-scale production of etoposide intermediates in a plant chassis. J. Am. Chem. Soc..

