# Supplementary material for: Transgenic Forsythia plants expressing sesame cytochrome P450 produce beneficial lignans
Source: Sci Rep. 2022 Jun 16;12:10152. doi: 10.1038/s41598-022-14401-9 (PMC9203787; doi:10.1038/s41598-022-14401-9)
Supplement: Supplementary file 1 — Supplementary Information. [file 41598_2022_14401_MOESM1_ESM.docx]

**Supplementary materials**

**TITLE**

Transgenic *Forsythia* plants expressing sesame cytochrome P450 produce beneficial lignans

**AUTHORS**

Tomotsugu Koyama^1^, Erika Matsumoto^1^, Toshimi Okuda^1^, Jun Murata^1^, Manabu Horikawa^1^, Naoki Hata^2^, Atsushi Okazawa^3^, Eiichiro Ono^4^, Honoo Satake^1^

**AFFILIATION**

^1^ Bioorganic Research Institute, Suntory Foundation for Life Sciences, Seikacho, Kyoto 619-0284, Japan

^2^ Department of Biological Resources Management, School of Environmental Science, The University of Shiga Prefecture, 2500 Hassaka-cho, Hikone, Shiga 522-8533, Japan

^3^ Graduate School of Life and Environmental Sciences, Osaka Prefecture University, 1-1 Gakuen-cho, Naka-ku, Sakai 599-8531, Japan

^4^ Suntory Global Innovation Center (SIC) Ltd., Research Institute, Soraku-gun, Kyoto, 619-0284, Japan

**Correspondence to:** Honoo Satake, Ph.D.

Bioorganic Research Institute, Suntory Foundation for Life Sciences,

Seikacho, Kyoto 619-0284, Japan

Phone: +81-70-2288-1385

Fax: +81-774-98-6262

E-mail: satake@sunbor.or.jp

**Legends to Supplementary Figures and Tables.**

**Supplementary Figure S1. Content of pinoresinol in the control and *Pro35S:CYP81Q1* plants.**

The leaves of plants after three rounds of vegetative propagation were applied to LC-MS analysis. The content of pinoresinol was calculated according to the areas of the peaks in the chromatograms of the standard curve of authentic pinoresinol. Six biological replicates of the measurement were averaged and statistically tested by Student’s *t*-test. Error bars indicate standard deviation of six biological replicates.

**Supplementary Figure S2. The full images of electrophoresed gels.**

Red rectangles were cut and pasted to prepare the Figure 2i, 2j, 3b and 3c.

**Supplementary Figure S3. Synthesis of 2´-ethoxysesamin.**

The reaction condition was followed the procedure reported previously^a^.

a Garcia, A. et al. Tetrahedoron 51, 8585-8598 (1995).

2´-ethoxysesamin: [α]D -13.0º (c 0.325, CHCl3), HRMS (ESI) calcd for C22H22O7Na [M+Na]+ 421.1258; found 421.1237. 1H and 13C NMR data show in Supplementary Table S2.

**Supplementary Table S1.　Primers used in this study.**

**Supplementary Table S2. ^1^H and ^13^C NMR data of 2´-ethoxysesamin.**


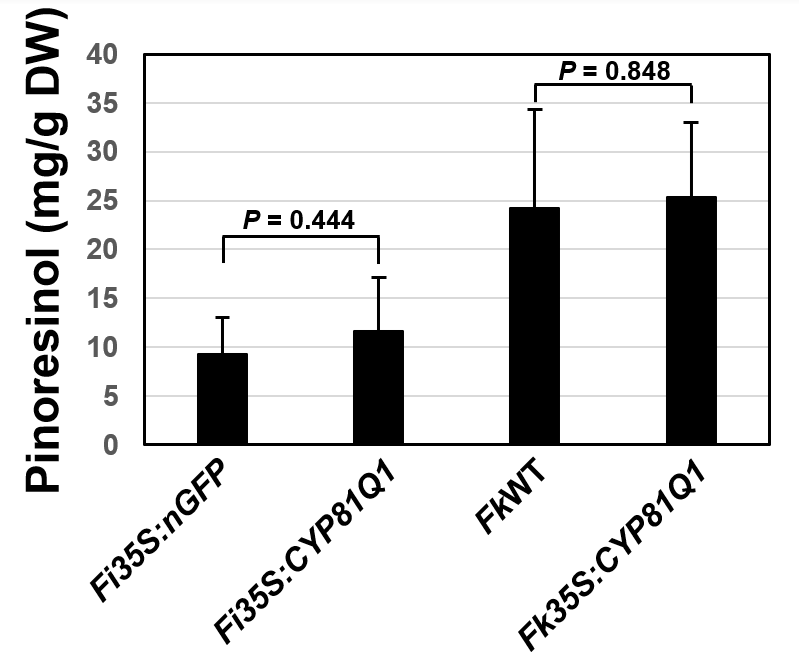


**Supplementary Figure S1. Content of pinoresinol in the control and *Pro35S:CYP81Q1* plants.**

The leaves of plants after three rounds of vegetative propagation were applied to LC-MS analysis. The content of pinoresinol was calculated according to the areas of the peaks in the chromatograms of the standard curve of authentic pinoresinol. Six biological replicates of the measurement were averaged and statistically tested by Student’s *t*-test. Error bars indicate standard deviation of six biological replicates.


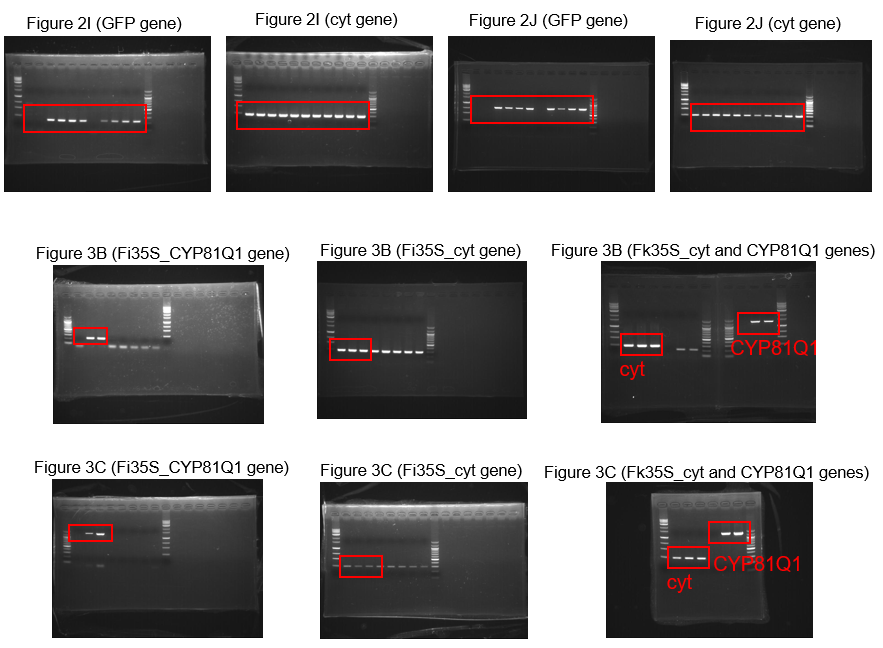


**Supplementary Figure S2. Full images of electrophoresed gels.**

Red rectangles were cut and pasted to prepare Figures 2i, 2j, 3b, and 3c.

**Supplementary Figure S3.** **Synthesis of** **2´-ethoxysesamin.**

The reaction conditions followed procedures reported previously ^a^.

^a^ Garcia, A. et al. *Tetrahedoron* **51**, 8585-8598 (1995).

**2´-ethoxysesamin**: [α]_D_ -13.0º (c 0.325, CHCl_3_), HRMS (ESI) calcd for C_22_H_22_O_7_Na [M+Na]^+^ 421.1258; found 421.1237. ^1^H and ^13^C NMR data shown in Supplementary Table S2.

**Supplementary Table S1. Primers used in this study.**

| Name | sequence |  |  | purpose |
| --- | --- | --- | --- | --- |
| ctDNAFw | AGTTCGAGCCTGATTATCCC | | | amplification of cytochrome gene (Figure 2 and 3) |
| ctDNARv | GCATGCCGCCAGCGTTCATC | | | amplification of cytochrome gene (Figure 2 and 3) |
| Tk0356-GFPfw | ATGGTGAGCAAGGGCGAG | | | amplification of GFP gene (Figue2) |
| Tk0357-GFPrv | TTACTTGTACAGCTCGTCCATGC | | | amplification of GFP gene (Figue2) |
| CYP81Q1-3Fw | GGGACCCATATTTTCCATCC | | | amplification of CYP81Q1 gene (Figure 3b_Fi_CYP81Q1) |
| CYP81Q1-3Rv | TCAAGATCGCACAACTTTCG | | | amplification of CYP81Q1 gene (Figure 3b_Fi_CYP81Q1) |
| CYP81Q1 Fw | ATGGAAGCTGAAATGCTATATTCAGCT | | | amplification of CYP81Q1 gene (Figure 3b_FkCYP81Q1, Figure 3c) |
| CYP81Q1Rv | AACGTTGGAAACCTGACGAAGTTTTTC | | | amplification of CYP81Q1 gene (Figure 3b_FkCYP81Q1, Figure 3c) |

**Supplementary Table S2****. ^1^H and ^13^C NMR data of 2´-ethoxysesamin.^a^**

|  | 2´-ethoxysesamin | | |
| --- | --- | --- | --- |
|  | ^1^H (ppm) | Additional data of ^1^H NMR^b^ | ^13^C (ppm) |
| 1 | - |  | 135.0 |
| 2 | 6.86 | 1H, br.s | 106.7 |
| 3 | - |  | 148.1 |
| 4 | - |  | 147.3 |
| 5 | 6.77 | 1H, d, *J* = 8.04 Hz | 108.3 |
| 6 | 6.81 | 1H, br.d, *J* = 8.04 Hz | 119.7 |
| 7 | 4.64 | 1H, d, *J* = 6.15 Hz | 85.6 |
| 8 | 2.92 | 1H, m | 54.6 |
| 9a | 4.19 | 1H, dd, *J* = 6.8, 9.0 Hz | 71.3 |
| 9b | 3.92 | 1H, dd, *J* = 3.8, 9.0 Hz |  |
| 10a, b | 5.94 | 2H, app.s | 101.2 |
| 1´ | - |  | 127.3 |
| 2´ | - |  | 139.7 |
| 3´ | - |  | 136.2 |
| 4´ | - |  | 148.8 |
| 5´ | 6.49 | 1H, d, *J* = 8.01 Hz | 102.1 |
| 6´ | 6.85 | 1H, d, *J* = 8.01 Hz | 118.3 |
| 7´ | 5.03 | 1H, d, *J* = 4.53 Hz | 82.9 |
| 8´ | 3.03 | 1H, m | 54.1 |
| 9´a | 4.33 | 1H, app.br.t, *J* = 9.1 Hz | 73.6 |
| 9´b | 4.01 | 1H, dd, *J* = 5.0, 9.1 Hz |  |
| 10´a, b | 5.9 | 2H, app.d, *J* = 6.4 Hz | 101.0 |
| 11´a | 4.35 | 1H, dq, *J* = 9.3, 7.1 Hz | 67.6 |
| 11´b | 4.28 | 1H, dq, *J* = 9.3, 7.1 Hz |  |
| 12´ | 1.37 | 3H, t, *J* = 7.1 Hz | 15.8 |

^a^ 800 MHz for ^1^H and 200 MHz for ^13^C NMR, Solvent: CDCl_3_ through alumina to remove an acid.

^b^ Number of hydrogens, Multiplicity (app.; apparently, br.; broad, s; singlet, d; doublet, t; triplet, q; quartet, m; multiplet), Coupling constants.
